# Treatment strategy for early-stage esophageal cancer

**DOI:** 10.1007/s11604-024-01547-x

**Published:** 2024-03-15

**Authors:** Katsuyuki Sakanaka

**Affiliations:** Department of Radiation Oncology and Image-Applied Therapy, Graduate School of Medicine, Kyoto University Graduate School of Medicine, 54 Shogoin Kawahara-cho, Sakyo-ku, Kyoto, 606-8507 Japan

**Keywords:** Early-stage esophageal cancer, Endoscopic resection, Radiotherapy, Surgery

## Abstract

Approximately 90% of esophageal cancers in Japan are squamous cell carcinomas, and they are often detected at earlier stages in Japan than in Western countries; superficial esophageal cancer without lymph node or distant metastasis comprises one-third of all esophageal cancers in Japan. Endoscopic resection is a minimally invasive treatment for superficial esophageal cancer; however, the risk of regional lymph node recurrence is negligible when it invades the submucosal layer or lymphovasculature. In such cases, surgical treatment is necessary to control regional lymph node recurrences, although the physical burdens and potential complications cannot be overlooked. Recently, clinical trials in Japan have shown promising clinical outcomes of organ preservation strategies. One strategy is initially performing endoscopic resection for superficial esophageal cancer, assessing the risk of lymph node metastasis based on pathological diagnosis for endoscopically resected specimens, and subsequently considering additional therapy (e.g., observation or prophylactic chemoradiotherapy)—another strategy aimed to cure superficial esophageal cancer through definitive chemoradiotherapy alone. The safety and efficacy of the two strategies have been evaluated in clinical trials, which showed that both organ preservation strategies are comparable to surgery in terms of overall survival. However, challenges include improving the accuracy of pretreatment endoscopic diagnosis and decreasing the local–regional recurrence after chemoradiotherapy. This review provides an overview of the latest standard treatment for early-stage esophageal cancer and its future perspectives.

## Introduction

In 2019, the incidences of esophageal cancer in Japan were 21,719 for males and 4663 for females, and the numbers of deaths related to esophageal cancer were 8864 for males and 2094 for females. Esophageal cancer accounts for 3% of cancer incidence and 3% of cancer-related deaths in Japan [1]. The predominant histological type is squamous cell carcinoma, constituting 90% of all cases, with smoking and alcohol consumption identified as risk factors. The comprehensive registry of esophageal cancer in Japan in 2015 demonstrated that the primary sites of tumor involvement were 4.6% in the cervical esophagus, 86% in the thoracic esophagus, and 8.5% at the esophagogastric junction, with the highest incidence in the thoracic esophagus [2]. At the time of diagnosis, the rates of clinical stages (Union for International Cancer Control [UICC] TNM, seventh edition) were 33.4% for stage IA (superficial disease without lymph node metastasis—early-stage esophageal cancer), 54.7% for stages IB–III (locally advanced), and 10.7% for stage IV (distant disease partially including locally advanced disease) [2]. The stage distribution indicates that a significant proportion of patients are detected at earlier stages in Japan than in the United States (local disease, 19%; regional disease, 34%; and distant disease, 44%) [3, 4]. This is probably attributed to the widespread use of upper esophagogastroduodenoscopy examinations for the early detection of gastric cancer in Japan, particularly in the high-quality screening programs of endoscopic specialists.

Surgical treatment has been the standard treatment for esophageal cancer from early to locally advanced stages. This procedure involves subtotal esophagectomy, regional lymph node dissection, and additional reconstruction of the digestive conduit using the stomach or colon. The surgical procedure applies even to early-stage patients, and the procedures are the same as those for advanced-stage patients. Esophageal cancer predominantly affects individuals in their 60–70 s, often in the elderly population, and the physical burden of esophagectomy and reconstruction is substantial, although surgical treatment of superficial esophageal cancer showed 73–86% in 5-year overall survival (OS) [4, 5].

According to a report of open esophagectomy for clinical T1N0M0 [6], the 30-day and in-hospital mortality rates for surgical resection of clinical stage I esophageal cancer were both 0%. However, the incidence of surgical complications was not low at 63% (36 of 57 cases). Among the surgical complications, the most prevalent was anastomotic leak at 32%, and recurrent laryngeal nerve paralysis, a relatively high-rate complication at 19%, primarily attributed to lymph node dissection around the recurrent laryngeal nerve, a part of the upper mediastinal lymph node group. In addition, pneumonia, a respiratory complication, was observed in 7% of cases. In a prospective cohort trial, thoracoscopic esophagectomy was considered less invasive and decreased the incidence of postoperative atelectasis compared with open esophagectomy; however, reoperation was more frequent in thoracoscopic esophagectomy [7]. In the long run, postoperative anatomical changes increase susceptibility to aspiration pneumonia from the reflux of contents such as digestive fluids and food from the reconstructed digestive tract. Even in the absence of esophageal cancer recurrence, the risk of death from other conditions should be considered. An intense desire exists to develop nonsurgical treatments from the perspective of organ preservation.

Two Japanese clinical trials recently showed that nonsurgical treatment strategies in clinically diagnosed superficial esophageal cancer without lymph node metastasis are comparable in OS to surgery [4, 8]. This review focuses on thoracic esophageal squamous cell carcinoma, a predominant disease in Japan, and describes the standard treatment for early-stage esophageal cancer, introducing recent clinical evidence using radiotherapy and future perspectives.

## Definition of early-stage esophageal cancer

**Fig. 1:**
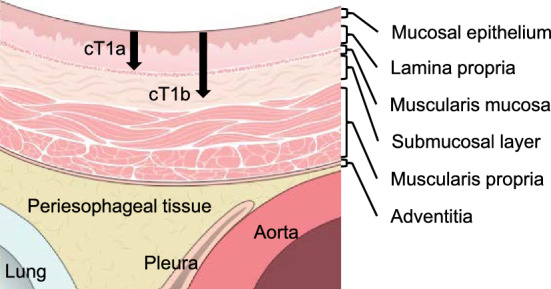
Schema of the esophageal wall

Esophageal cancer originates from the mucosal epithelium (EP) and invades the lamina propria (LPM), muscularis mucosae (MM), submucosal layer, muscularis propria, and adventitia of esophagus (Fig. 1). The Japanese Classification of Esophageal Cancer (12th edition) defined a primary esophageal cancer remaining within the esophageal mucosa (EP, LPM, and MM) as T1a, an early carcinoma of the esophagus irrespective of lymph node metastasis, whereas those extending to the submucosal layer were termed T1b, a superficial carcinoma of the esophagus, irrespective of lymph node metastasis [9]. T1b involves submucosal layer invasion (SM1, upper third of the submucosal layer; SM2, middle third of the submucosal layer; and SM3, lower third of the submucosal layer) [9]. The classification of the three submucosal layers (SM1, SM2, and SM3) is based on a specimen of surgical resection. In endoscopically resected specimens, SM1 is defined as infiltration up to 200 μm from the MM, with deeper levels categorized as SM2 and SM3 undefined in endoscopically resected specimens [9]. The current review includes clinically diagnosed esophageal cancer extending from the EP to the SM without any lymph node metastasis.

**Fig. 2:**
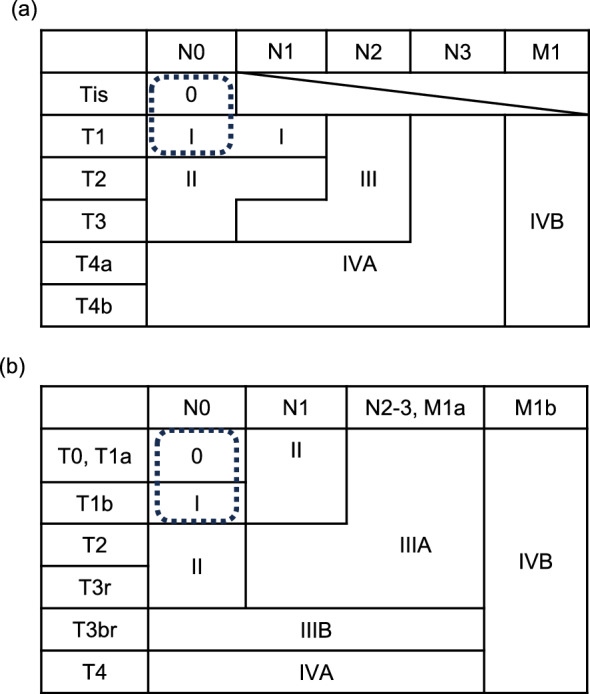
Clinical staging of esophageal squamous cell carcinoma based on (**a**) the Union of International Cancer Control TNM classification, eighth edition, and (**b**) the Japanese Classification of Esophageal Cancer, 12th edition. The area enclosed by the dashed line indicates the clinical stage described in this review

The clinical and pathological staging of esophageal cancer is classified based on the UICC TNM staging system, an international standard (Fig. 2a), and the Japanese Classification of Esophageal Cancer (Fig. 2b). Differences are noted between these two classification systems in assessing the clinical depth of invasion, lymph node metastasis numbers, and diagnosis of regional lymph nodes. This review uses the staging classification for squamous cell carcinoma based on the Japanese Classification of Esophageal Cancer, 12th edition, and focuses on esophageal cancer of clinical stages 0 (cT1aN0M0) and I (cT1bN0M0) (Fig. 2b).

## Clinical diagnosis of the depth of invasion and lymph node metastasis

Endoscopic examination is key for clinical diagnosis of the depth of invasion. The depth of invasion differentiates superficial esophageal cancer into EP/LPM, MM/SM1, and SM2 or deeper. Advances in endoscopic equipment, such as magnifying endoscopy, narrow band imaging, and endoscopic ultrasonography, have improved diagnostic capabilities; however, the concordance rate between clinical and pathological diagnosis for depth of invasion is not high. Even when clinically diagnosed as MM/SM1 cancer before treatment, 27.4%–55.2% of the patients were pathologically EP/LPM cancer, which was likely to be cured by endoscopic resection (overdiagnosed), but conversely proved to be pathologically SM2 cancer, indicating deeper involvement before treatment, were found in 15.5–27.9% of the patients (undertreatment) [10].

Similar to endoscopic diagnosis for depth of invasion, the diagnostic accuracy of lymph node metastasis using contrast-enhanced computed tomography (CT) is not high. Generally, cervical to abdominal lymph nodes along with the esophagus are considered potentially metastatic lymph nodes if their short axial diameter is 5–10 mm or more; however, there are several cases where lymph node metastasis is absent, leading to false-negative results. When using a criterion of ≥ 10 mm, the sensitivity and specificity of contrast-enhanced CT are 30–60% and 60–80%, respectively [11, 12]. To assess the degree of fluorodeoxyglucose (FDG) accumulation, which is considered more accurate than size-dependent CT diagnosis, the sensitivity and specificity of FDG-positron emission tomography are still 51–66% and 84–100%, respectively [12, 13]. The clinical diagnosis of the depth of invasion and lymph node metastasis includes limitations. We need to treat this population considering these uncertainties.

## Nonsurgical treatments for early-stage esophageal cancer

### Clinically diagnosed EP to LPM tumors

Squamous cell carcinoma of the esophagus within the mucosal layer (cT1a), such as EP or LPM invasion, has been found to have a low frequency of clinical or latent lymph node metastasis based on the report of surgical resection specimens [14]. Therefore, minimally invasive endoscopic resection is a standard curative treatment for this population. However, the circumferential extent (for > 3/4 circumferential lesions) or longitudinal length (> 5 cm) of the mucosal defect after endoscopic resection is associated with the development of esophageal stenosis [15–17]. The Guidelines for Diagnosis and Treatment of Carcinoma of the Esophagus 2022, edited by the Japan Esophageal Society, recommended surgical resection or chemoradiotherapy for whole circumferential or longitudinal lengths of tumors of > 5 cm [18].

### Clinically diagnosed MM to SM1 tumors

The development of endoscopic treatments enables complete endoscopic resection of primary esophageal cancer, even in esophageal tumors with SM invasions, provided that the deepest part remains around the middle layer of the SM. However, cancers infiltrating pathologically diagnosed MM (cT1a) or SM1 (cT1b) tend to exhibit lymph node metastasis in 10–40% of cases as the depth increases [19]. As described earlier, the discordance rate between clinical and pathological diagnoses for the depth of invasion is substantial. Furthermore, lymphovascular invasion is a significant risk factor for lymph node recurrences in esophageal cancer [20–22], whose diagnosis requires endoscopically or surgically resected specimens.

**Fig. 3:**
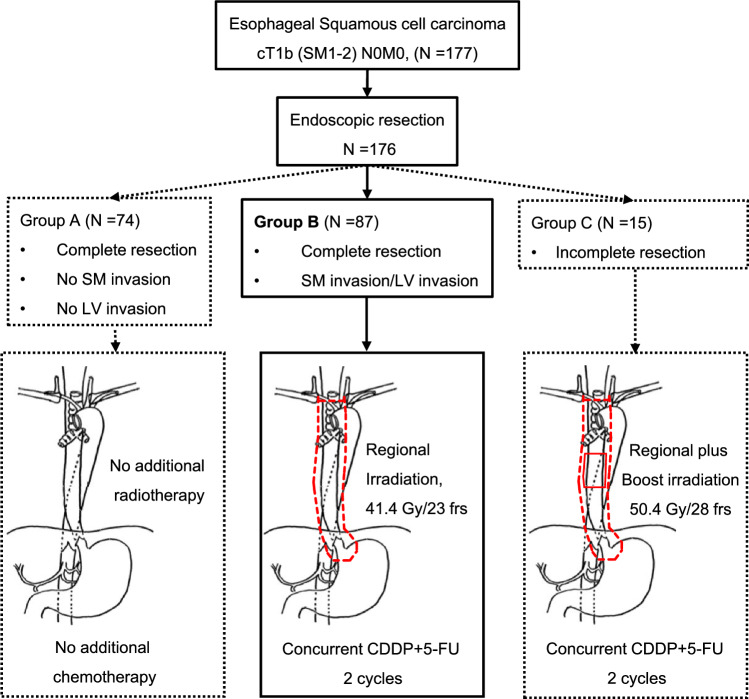
JCOG0508 treatment algorithm and consort diagram. *SM* submucosal layer, *LV* lymphovascular, *frs* fractions, *CDDP* cisplatin, *5-FU* 5-fluorouracil

Retrospective studies have suggested the effectiveness of endoscopic resection plus additional chemoradiotherapy for patients deemed at high risk of recurrence [23], and this treatment strategy may be comparable to additional surgery [24, 25]. Then the Japanese multi-institutional single-arm confirmatory phase II trial (JCOG0508) addressed the effectiveness and safety of this treatment strategy [8, 26]. JCOG0508 adopted initial endoscopic resection and additional chemoradiotherapy only to patients deemed at high risk of lymph node metastasis based on the pathological diagnosis of the depth and vascular invasion, avoiding potential over- or undertreatment where surgery or chemoradiotherapy may be necessary or not. Clinically diagnosed cT1N0M0 esophageal cancer with submucosal invasion (SM1–SM2) was eligible. Within 1 month of registration, the enrolled patients underwent endoscopic resection. The pathological findings of endoscopic specimens determined the additional treatments: patients with completely resected mucosal cancer (EP, LPM, and MM) without vascular invasion underwent observation (Group A), those with completely resected mucosal cancer but positive vascular invasion or SM invasion received additional prophylactic chemoradiotherapy using 41.4 Gy in 23 fractions for regional lymph nodes (primary analysis target) (Group B), and those with positive deep margins after endoscopic resection underwent additional definitive chemoradiotherapy using 50.4 Gy in 28 fractions to tumor bed plus 41.4 Gy to regional lymph nodes (Group C). Three-dimensional conformal radiotherapy using CT simulation was mandatory, using multiple fields, particularly in the middle and lower esophagus (Fig. 3).

JCOG0508 trial enrolled 177 patients from December 2006 to July 2012. One patient withdrew consent before endoscopic treatment, and six patients discontinued protocol treatment (four in Group B and two in Group C); ultimately, 74 patients underwent no additional treatment (Group A), 83 received prophylactic chemoradiotherapy (Group B), and 13 received definitive chemoradiotherapy (Group C). The results concluded that the 3-year OS in the primary analysis target (Group B) was 90.7% (90% confidence interval [CI] 84.0–94.7%), and the 3-year OS for all enrolled patients, including observation (Group A) and definitive chemoradiotherapy (Group C), was 92.6% (90% CI 88.5–95.2%), both exceeding the lower limit of the CI (80%) predefined before the trial, achieving observed outcomes comparable to surgical resection. No serious adverse events occurred with endoscopic treatment in JCOG0508; however, one patient in Group B experienced esophageal stenosis (grade 3, 0.6%), preventing the addition of prophylactic chemoradiotherapy. Grade 3 or higher adverse events due to chemoradiotherapy included neutropenia (22.9%), hyponatremia (7.3%), esophagitis (4.2%), and anorexia (7.3%), with late cardiac toxicity observed in two cases (2.1%). Recurrence occurred in 15 patients (8.5%): 1, 10, and 4 in Groups A, B, and C, respectively. Lymph node recurrence occurred in 11 patients (2 cervical, 10 thoracic, and 4 abdominal lymph nodes), and distant organ metastasis was observed in 5 patients (with overlap). Seven patients underwent salvage surgery, and two survived. The follow-up results of ≥ 5 years suggested that venous invasion, a single course of chemotherapy, and pathological SM2 with lymphovascular invasion are risk factors for recurrence [26].

The Guidelines for Diagnosis and Treatment of Carcinoma of the Esophagus 2022 mentions the initial endoscopic resection plus chemoradiotherapy for patients at high risk of recurrence: there is evidence to recommend esophagectomy or chemoradiotherapy as an additional treatment in patients identified as having a pT1a-MM lesion with positive vascular invasion or a pT1b-SM lesion after endoscopic treatment for superficial esophageal cancer [18].

### Clinically diagnosed SM2 or deeper tumors

Clinically diagnosed SM2 or deeper tumors are ineligible for endoscopic resection because of the high risk of incomplete resection or perforation of the esophageal wall with endoscopic procedures. The standard treatment for this population has been surgery; however, definitive chemoradiotherapy has been suggested as a less invasive alternative, showing comparable efficacy to surgery. In a single-arm phase II trial (JCOG9708) investigating the effectiveness of definitive chemoradiotherapy of locally 60 Gy in 30 fractions with concurrent cisplatin and 5-fluorouracil for cT1N0M0 esophageal cancer, a complete response rate of 87.5% and 5-year OS of 75.5% demonstrated favorable treatment outcomes [27]. Based on this background, a non-inferiority trial (JCOG0502) was planned for definitive chemoradiotherapy versus surgery for cT1b of SM2 or deeper esophageal cancer.

**Fig. 4:**
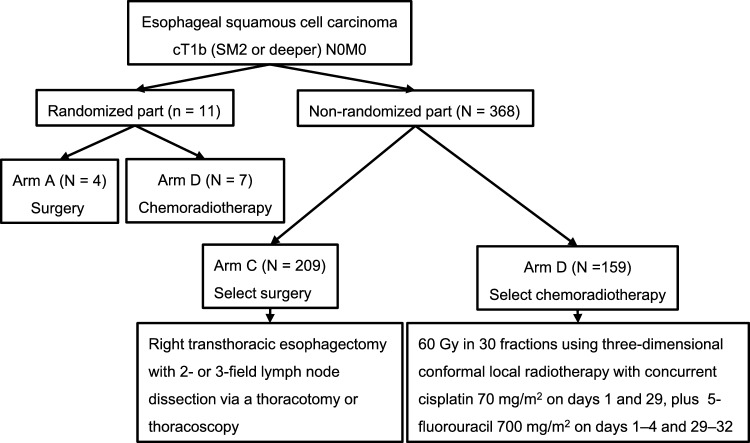
JCOG0502 consort diagram

JCOG0502 included patients with T1b (SM2 or deeper) N0M0 thoracic esophageal cancer histologically diagnosed as squamous cell carcinoma, adenosquamous carcinoma, and basaloid carcinoma. Eligible patients were aged 20–75 years, with no prior treatment for esophageal cancer, no pretreatment history of endoscopic resection for esophageal cancer, and no pretreatment history of chemotherapy, radiotherapy, or hormonal therapy for other cancers. Patients who consented to randomization were assigned to surgery (Group A) or chemoradiotherapy (Group B), whereas non-consenting patients to randomization selected the treatments by themselves—surgery (Group C) or chemoradiotherapy (Group D)—and were followed. In Groups A and C, transthoracic esophagectomy with two- to three-field lymph node dissection via thoracotomy or thoracoscopy was performed. In Groups B and D, chemoradiotherapy consisted of 5-fluorouracil 700 mg/m^2^ on days 1–4 and 29–32, cisplatin 70 mg/m^2^ on days 1 and 29, and local radiotherapy at 60 Gy in 30 fractions (5 days/week) (Fig. 4).

The primary endpoint was OS for Groups A and B, with secondary endpoints including OS for Groups C and D, adverse events, and progression-free survival (PFS) in each group and complete response rate and esophagectomy-free-survival in Groups B and D; however, because of poor accrual in the randomized part (Groups A and B), the randomization part was terminated in February 2011, and the non-randomized part only continued: a total of 379 patients (11 patients in the randomized part and 368 in the non-randomized part) were enrolled between December 2006 and February 2013. A comparison was made in the non-randomized part, excluding Groups A and B patients in the final analysis of JCOG0502 [4].

Patient characteristics showed no significant differences in the median age, sex, performance status, primary tumor site, histological type, tumor length (≤ 4 vs. > 4 cm), and multiple lesions. The 3-year median OS rates in the non-randomized portion was 94.7% (95% CI 90.6–97.0) for Group C and 93.1% (95% CI 87.9–96.1) for Group D, with the 5-year median OS rates being 86.5% (95% CI 81.0–90.5) for Group C and 85.5% (95% CI 78.9–90.1) for Group D. The adjusted hazard ratio (HR) of OS was 1.052 (95% CI 0.674–1.640), indicating statistical non-inferiority of chemoradiotherapy compared with surgery. The 3-year PFS rates were 84.1% (95% CI 78.4–88.4) for Group C and 76.1% (95% CI 68.7–82.0) for Group D, whereas the 5-year PFS rates were 81.7% (95% CI 75.7–86.3) for Group C and 71.6% (95% CI 63.9–78.0) for Group D. The adjusted HR of PFS was 1.478 (95% CI 1.010–2.162), favoring surgery. The complete response rate in the chemoradiotherapy group was 87.3% (95% CI 81.1–92.1), which was comparable to the previous single-arm phase II trial of chemoradiotherapy for cT1N0M0 esophageal cancer (JCOG9708) [27].

Safety assessment revealed acute adverse events in the chemoradiotherapy group, including Grade 3–4 leukopenia (11.4%), neutropenia (11.4%), esophagitis (10.1%), and febrile neutropenia (1.9%). Late adverse events included esophagitis (0.6%), pneumonia (1.9%), pleural effusion (2.5%), and myocardial ischemia (3.2%). No fistula formation or pericardial effusion occurred, indicating the feasibility of safe treatment. The common grade 3–4 postoperative complications in the surgery arm were increased levels of alanine transaminase (20.8%), aspartate transaminase (8.7%), and total bilirubin (8.7%); pneumonia (7.7%); anastomotic leakage (6.3%); and recurrent nerve paralysis (2.9%).

Fifty-six patients in Group C and 57 patients (35.8%) in Group D underwent subsequent treatment: endoscopic resection for 16 patients in Group D, chemotherapy for 48 patients in Group C and 24 patients in Group D, surgery for 10 patients in Group C and 21 patients in Group D, and radiotherapy for 6 patients in Group C and 7 patients in Group D. The 3- and 5-year esophagectomy-free survival rates of patients in Group D were 88.7% (95% CI 82.6–92.7) and 80.4% (95% CI 73.3–85.8), respectively.

In conclusion, esophagectomy and chemoradiotherapy demonstrated effectiveness and safety for cT1b (SM2 or deeper) esophageal cancer. Chemoradiotherapy showed non-inferiority in OS compared with esophagectomy despite non-randomized part analysis, suggesting that chemoradiotherapy is one of the standard treatments for cT1b (SM2 or deeper) esophageal cancer; however, the PFS of chemoradiotherapy is inferior to that of surgery.

## Future directions

JCOG0508 showed that endoscopic resection for cT1b (SM1–SM2) N0M0 plus additional chemoradiotherapy for patients with high-risk factors (pSM or lymphovascular invasions) had comparable oncological outcomes to adding surgery. As an additional treatment for high-risk patients, esophagectomy and chemoradiotherapy are both equally recommended at this time, and it is impossible to determine which one to recommend now. A phase III trial is currently ongoing, which compares additional surgery or chemoradiotherapy for patients deemed at high risk of recurrence after endoscopic resection [28]. This trial may conclude which is better as an additional treatment for high-risk patients after endoscopic resection.

**Fig. 5:**
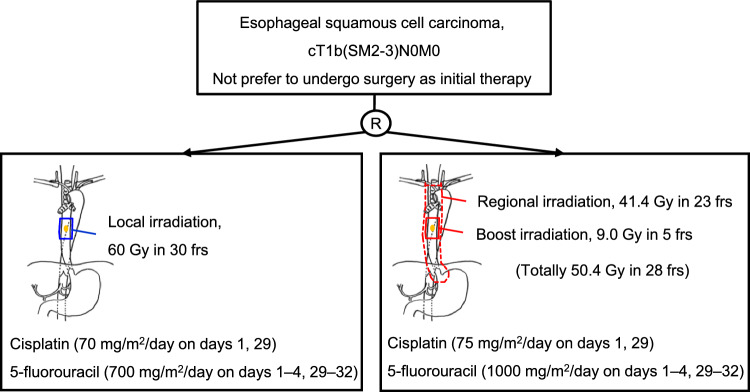
JCOG1904 trial schema. *SM* submucosal layer, *frs* fractions

JCOG0502 established that chemoradiotherapy is comparable in OS to surgery for cT1b (SM2 or deeper) N0M0 esophageal cancer. However, JCOG0502 showed that chemoradiotherapy is associated with a higher incidence of esophageal mucosa or regional lymph node recurrences than surgical therapy. A phase III randomized controlled trial (JCOG1904) comparing the local field with additional prophylactic irradiation in chemoradiotherapy for clinical T1bN0M0 esophageal cancer is underway [29]. This trial compares the regimens of chemoradiotherapy using 50.4 Gy in 28 fractions plus regional lymph node irradiation of 41.4 Gy in 23 fractions with the dose-intensified cisplatin and 5-fluorouracil to the chemoradiotherapy regimen of JCOG0502 in patients with clinical T1b (SM2 or deeper) N0M0 esophageal cancer who do not prefer to undergo surgery as initial therapy (Fig. 5). The addition of prophylactic irradiation and an escalated dosage of chemotherapy may prevent regional lymph node recurrences and systemic metastasis and improve the PFS, which was inferior in chemoradiotherapy to surgery in JCOG0502.

## Conclusion

The physical burden of surgical treatment is not negligible; however, surgery has been a standard treatment for early to advanced esophageal cancer. Less toxic treatments that achieve organ preservation were explored. Endoscopic resection plus chemoradiotherapy or definitive chemoradiotherapy has become one of the standard treatments for early-stage esophageal cancer in the Japanese clinical trials (JCOG0502 and JCOG0508). Internationally, the incidence of esophageal cancer at an early stage remains relatively low. It is anticipated that as early diagnosis of esophageal cancer becomes more prevalent by widespread endoscopic examination, especially in Asia, evidence from JCOG0502, JCOG0508, and ongoing clinical trials will be increasingly valuable in the future.
